# HIV vaccines beyond COVID‐19: merits of trust

**DOI:** 10.1002/jia2.25742

**Published:** 2021-05-17

**Authors:** James G Kublin

**Affiliations:** ^1^ Fred Hutchinson Cancer Research Center University of Washington Seattle USA

**Keywords:** HIV, TB, COVID‐19, vaccine, translational medical research, citizen science

As contagion of sickness makes sickness, contagion of trust can make trust. In Distrust of Merits, Marianne Moore [1]

The astonishing success of the SARS‐CoV‐2 vaccines reveals important aspects of the vaccine development process that should be applied to future pandemics, as well as to those that have been with us for decades, or, in some cases, millennia. This Viewpoint will focus on how HIV and TB vaccine research will change based on what we have learned about public health and vaccine development over the course of the SARS‐CoV‐2 pandemic, particularly in the areas of funding, biotechnology discoveries, workforce capacity and community participation and equity.

The COVID‐19 vaccine platforms developed over the past year have relied upon decades of prior vaccine research and development [2, 3], most notably the collaborative efforts between government, academia and industry working together on HIV vaccine research. These novel discoveries were instrumental in bringing us safe, efficacious COVID‐19 vaccines within a year [4]. Government funding of these basic science discoveries enabled the technological advances that researchers could pull off the shelf in their race to develop a SARS‐CoV‐2 vaccine (e.g. mRNA vaccine platforms, the isolation and characterization of monoclonal antibodies as well as new diagnostic tools). Even more discoveries will likely be applied to further preparedness for future pandemics.

The significance of prior discoveries in pandemic vaccine development shows that continued funding is needed for investigations that might not always be on a product‐development critical path. Individual investigators achieved basic discoveries, making subsequent advances to develop COVID‐19 vaccines possible in a year; then multinational clinical trial networks initially established for HIV vaccine research enabled Phase 3 efficacy testing. Although some have raised concerns about duplication of efforts, there has been intentional duplication of platforms and immunogens to achieve the development and production of COVID‐19 vaccines, and much will be learned from comparisons between platforms and vectors [5]. Pursuing scientific questions about how these vaccines worked – questions that are not focused directly on product development – will reap long‐term benefits for future vaccines.

It may be years before we identify the most important scientific contributions from the responses to the SARS‐CoV‐2 pandemic to the future of HIV and TB vaccine research. The world now knows that nucleic acid and viral vector approaches to immunization (e.g. mRNA vaccines) can be very effective [6]; yet, studying mechanisms responsible for the high levels of protection may direct us in unanticipated ways. If neutralizing antibodies to SARS‐CoV‐2 are as critical as expected, what innate and cellular components are mediating these responses, ensuring specificity and durability? Which preclinical models were best at predicting these correlates? Are the immune responses that control virus in different anatomic sites successful (or not) in ensuring that further transmission does not occur? How does the heterogeneity of these responses shed light on the spectrum of disease pathology? Is there a role for therapeutic vaccination to decrease long‐term sequelae of infection? The tools used to develop the SARS‐CoV‐2 vaccines were not necessarily new, but they are newly proven to be effective; so science may leapfrog years of trial and error in answering these questions.

While more people than ever have closely followed science during this pandemic, it has also highlighted the difficulty of effective science reporting. We have witnessed the often‐confusing consequences of “publishing” data by press release. Moreover, accuracy suffers from the media (including social media) and consumers’ bad‐news bias [7]. In contrast, peer review in scientifically rigorous journals engages multiple co‐authors, especially for large clinical trials, who can speak with authority and clarity, regardless if the news is good or “bad.” Science reporting matters, for many reasons, not least because an informed public will support investment into basic and applied research. Going forward, our informed citizenry needs to be continuously engaged, with the scientific community partnering with advocates and consumers to explain results and maintain the momentum of the past year.

Scientific innovation must continue with open, creative “citizen science” [8, 9, 10, 11, 12] that will attract generations of investigators. Future investigators are hungry to be trained and need opportunities and quality mentoring. The pedagogic efforts early in the pandemic led to an unprecedented expansion in science literacy among the general public, and we have a unique opportunity to continue increasing the research literacy of civil society at large. Although public attention will likely wane, building on current interest to expand the workforce is critical. More than ever, attention to early science education and early stage investigators across discipline deserves attention. Future investigators, their interest in biotechnology piqued by living through the pandemic, will be hungry to be trained and they will need opportunities and quality mentoring. Pandemic preparedness will rely on the interconnected work of virology, microbiology and public health sciences, including strong transnational animal and human disease surveillance.

Born of necessity, the pandemic has shown that virtual engagement can be more inclusive and that democratization and sharing of data can be a knowledge accelerator. The response to the current pandemic has given us a lens on global and national strengths, disparities and opportunities. The scientific community has rallied to address pandemic challenges [11, 12]; the media and advocates and community representatives demanded clarity on methods and timelines and involvement in the process; academic and corporate leadership pushed to ensure more rapid regulatory pathways and commercial support. There were also lost opportunities: reluctance and caution imposed unnecessary barriers to optimal success. We were reminded that health disparities reflected impediments to the prevention and therapeutic interventions that save lives, and that the stigma of infection has persisted throughout the COVID‐19 pandemic [13, 14]. We have much room to improve.

We have also learned that addressing a pandemic works best when it keeps equity at the forefront. As HIV community advocates have said since the beginning of that pandemic, we have better outcomes when participation in clinical trials includes those who are most impacted. Effectively combating public health crises requires engaging all communities throughout the scientific process [15], and maintaining detailed metrics to chart success and failure to that end [16]. The success of data‐driven representation of racial and ethnic minorities and other underserved groups in COVID‐19 clinical research shows that equitable participation is feasible when all involved make it a top priority, which requires authentic community engagement [17]. Pandemic responses also demand rapid and accurate public data, and assistance is needed from a globally engaged citizenry of millions of people to keep the momentum of trust.

Over this incredibly challenging year, we have made advances in vaccine research and development that were nothing short of extraordinary. But we must not be complacent. The progress on COVID‐19 vaccines should lead to progress on HIV and TB vaccines. Some countries have seen fewer people die of COVID‐19 than HIV and TB this past year, so the continued importance of these older pandemics cannot be ignored. Like SARS‐CoV‐2, multidrug‐resistant TB also circulates on airplanes, in restaurants, apartment buildings and hospital waiting rooms worldwide. We now have tools to make vaccine advances against these other pathogens as well. The response to the SARS‐CoV‐2 pandemic has shown what a singular focus will achieve. Global attention to these pandemics must be a lasting, moral obligation.

## Competing interests

The authors have declared no conflict of interest.
